# Temporal variations of human and animal *Rotavirus A* genotypes in surface water used for drinking water production

**DOI:** 10.3389/fmicb.2022.912147

**Published:** 2022-08-09

**Authors:** Takayuki Miura, Syun-suke Kadoya, Hiroyuki Takino, Daisuke Sano, Michihiro Akiba

**Affiliations:** ^1^Department of Environmental Health, National Institute of Public Health, Wako, Japan; ^2^Department of Civil and Environmental Engineering, Tohoku University, Sendai, Japan; ^3^Department of Urban Engineering, The University of Tokyo, Bunkyo, Japan

**Keywords:** rotavirus, massive parallel sequencing, molecular epidemiology, surface water, drinking water source

## Abstract

Rotavirus is a major cause of gastroenteritis among infants and children. In this study, nested PCR assays were developed to amplify partial regions of the VP7, VP4, and VP6 genes of *Rotavirus A* (RVA) for amplicon-based Illumina MiSeq sequencing to investigate RVA genotypes in environmental water samples. Eight sets of inner primers were first designed and screened for use in the nested PCR assays, and four sets of them could produce amplicons. Six sets of outer primers were then designed and combined with the four sets of inner primers that worked. The assays were evaluated for sensitivity using raw water samples collected from one drinking water treatment plant between April 2019 and March 2020 (Sample Set 1; *N* = 12) and seven DWTPs between 2018 and 2020 (Sample Set 2; *N* = 18). In total, 43 amplicons from Set 1 were sequenced and diverse sequences from human, porcine, bovine, equine, and feline RVA were observed. Human G8, G3, and G2 genotypes were obtained, with G8 predominant (relative abundance, 36–87%) in samples taken during the rotavirus epidemic season between April and June. Porcine G5, G11, and G4, and bovine G10 and G6 genotypes were also detected. VP4 sequence analysis revealed that the human P[8] genotype was present throughout the year, whereas P[4] and P[9] were present only in the epidemic season. The vaccine strains P[5] and P[8] (RotaTeq^®^) were also detected. Our approach enables the identification of prevalent human and animal RVA genotypes and their host species that potentially caused fecal contamination in water sources.

## Introduction

Environmental water is often contaminated with wastewater from cities or farms and may contain both human and animal pathogens. Rotaviruses are viral agents that cause severe diarrheal disease and death in infants, children, and young animals (Dhama et al., 2009) and have been detected in both surface and groundwater sources (Miura et al., 2019, 2021; Sylvestre et al., 2021). Rotaviruses have been identified as potential reference pathogens in the WHO Guidelines for Drinking-Water Quality (WHO, 2017). To evaluate the risk from rotaviruses and maintain the safety of drinking water, a better understanding of the rotavirus genotypes that are present in water sources is required.

Rotaviruses belong to the genus *Rotavirus* in the family *Reoviridae* and have a non-enveloped structure with triple-layered icosahedral capsids that are approximately 100 nm in diameter including spikes. *Rotavirus* is currently divided into nine groups (*Rotavirus A* − *D*, *F* – *J*; ICTV, 2020). *Rotavirus A* − *C* has been found in both humans and animals (Estes and Greenberg, 2013) and *Rotavirus A* (RVA) is one of the important etiological agents of diarrhea in humans. The rotavirus genome consists of 11 double-stranded (ds)RNA segments that encode six structural proteins (VP1–4, VP6, and VP7) and six nonstructural proteins (NSP1–6; Estes and Greenberg, 2013). Based on the RNA sequences that encode VP7 (G: glycoprotein) and VP4 (P: proteinase-sensitive protein), RVA has been classified into the G and P genotypes (Estes and Greenberg, 2013). Recently, a whole-genome classification system was established based on 11 segments: Gx-P[x]-Ix-Rx-Cx-Mx-Ax-Nx-Tx-Ex-Hx, representing the VP7-VP4-VP6-VP1-VP2-VP3-NSP1-NSP2-NSP3-NSP4-NSP5/6 genotypes, respectively (“x” indicates the number of corresponding genotypes; Matthijnssens et al., 2011).

To detect and identify RVA strains in clinical samples, reverse transcription PCR (RT-PCR) assays have been designed for VP7 [e.g., Beg9/End9 primers (Gouvea et al., 1990)], VP4 [e.g., Con3/Con2 primers (Gentsch et al., 1992); Con1/Con4 primers (Fischer et al., 2003)], and VP6 [e.g., VP6-F/VP6-R primers (Iturriza Gómara et al., 2002)]. Nested PCR assays can be applied to sewage and river water samples using inner primers combined with outer primers that were developed for clinical sample analysis (Villena et al., 2003; Fumian et al., 2011; El-Senousy et al., 2020). The prevalence of G and P genotypes in water samples can be determined using nested PCR assays and amplicon-based Sanger sequencing. However, RVA concentrations in drinking water sources are generally too low to obtain amplicons from water using nested PCR assays.

*Rotavirus A* contamination in surface waters has previously been investigated using real-time PCR targeting the NSP3 gene (Prevost et al., 2015; Miura et al., 2019; Brooks et al., 2020) or the VP6 gene (Mackowiak et al., 2018), and median or mean concentrations ranging from 10^2^ to 10^5^ copies/L have been reported. A high prevalence (86%) and high concentrations (up to 5.5 log_10_ copies/L) of RVA were observed in surface water samples from 21 drinking water treatment plants (DWTPs) in Japan, suggesting RVA contamination with strains from livestock and vaccinated humans (Miura et al., 2019). Two rotavirus vaccines, Rotarix^®^ (GlaxoSmithKline Biologicals, Rixensart, Belgium) and RotaTeq^®^ (Merck & Co., Inc., Kenilworth, NJ, United States) introduced in Japan in November 2011 and July 2012, respectively, have been routinely given since October 2020 (Tsugawa et al., 2021). The VP6 gene that is specific to RotaTeq^®^ was detected in sewage samples using real-time PCR (Gautam et al., 2014; Ito et al., 2021), raising questions about the impact of vaccine strains as well as wild-type strains on water contamination with RVA. Amplicons from previously reported nested PCR assays for VP7 and VP4 were commonly in the range 663–881 bp (Tort et al., 2015; Imagawa et al., 2020); however, shorter amplicons (200–300 bp) are preferable for next-generation sequencing (NGS) of RVA strains in water samples.

In this study, inner and outer primer sets for use in the nested PCR assays for amplifying partial regions of the VP7, VP4, and VP6 genes were newly designed to be suitable for NGS. The primer sets were tested for sensitivity using viral RNA samples from water samples collected at DWTPs. The amplicons obtained were subjected to NGS and the diversity and seasonal variations of the RVA genotypes were investigated.

## Materials and methods

### Primer design

To design primers specific to partial regions of the VP7, VP4, and VP6 genes in human or animal RVA, complete genome sequences were obtained from GenBank.^1^ The keywords “rotavirus,” “A,” “VP7, VP4, or VP6,” and “complete” were used to obtain 33 and 16 VP7, 25 and 23 VP4, and 24 and 14 VP6 sequences for human and animal RVA, respectively. The sequences were aligned using ClustalW in the MEGA X software (Kumar et al., 2018) and conserved regions were selected visually. Consensus sequences were generated for human or animal VP7, VP4, and VP6 using the “Extract Consensus Sequence” function in CLC Genomics Workbench ver. 11 (Qiagen, Hilden, Germany), and primer sequences were selected from the sequences with Primer3Plus (Untergasser et al., 2012). Eight sets of inner primers (expected amplicon sizes, 229 to 272) were designed and evaluated for their potential to amplify a target gene from viral RNA (screening of designed primers). Based on the result of screening that four inner primer sets could produce amplicons, six sets of outer primers (expected amplicon sizes, 299–462 bp) were then designed for use with the four inner primer sets.

### Viral strain

Murine norovirus (MNV) strain S7-PP3 was provided by Dr. Yukinobu Tohya (Nihon University, Japan) for use as a process control to evaluate the virus recovery efficiencies from surface water samples. MNV was cultured using RAW 264.7 cells (ATCC TIB-71) following the methods described by Kitajima et al. (2010) and viruses were recovered and purified using a centrifugal ultrafiltration device (Miura et al., 2021). The purified MNV stock solution was maintained at −80°C until further use.

### Surface water samples

Viral RNA from two sets of surface water samples was used to select and evaluate the designed primer sets. Grab samples for Set 1 (*N* = 12) were collected monthly between April 2019 and March 2020 at DWTP E in the Kanto region. The grab samples were collected from a sampling tap connected to the raw water transmission main to the receiving tank in the plant. The plant, which treats river water, is downstream of several highly-populated cities and livestock farms. The pH, electrical conductivity (EC), and turbidity of the collected samples were in the ranges 7.1–7.5, 11–26 mS/m, and 1.3–34 turbidity units of polystyrene latex (TU-PSL), respectively.

To test the sensitivity of the nested PCR assays, Set 2 (*N* = 18) was used to ascertain the RVA levels (range, 3.4–6.3 log_10_ copies/L, determined by quantifying the NSP3 gene). The grab samples for Set 2 were collected from seven DWTPs (DWTP A, C, D, G, O, R, and U), which obtained raw water from rivers or reservoirs between 2018 and 2020. The DWTPs are located downstream of at least one wastewater treatment facility. Raw waters of these plants were also thought to contain effluents from wastewater treatment facilities, livestock farms, and the feces of wild animals (i.e., deer and boar). The pH, EC, and turbidity values of the collected samples were in the ranges 6.8–8.0, 9.1–34 mS/m, and 0.3–219 TU-PSL, respectively.

Water samples (5–6 l) were transported to the laboratory at the National Institute of Public Health within 2 days under cool conditions (0–10°C) and processed within 24 h of arrival.

### Preparation of viral RNA

#### Virus concentration

Water samples were filtered (Miura et al., 2019) and concentrated *via* the adsorption-elution method (Katayama et al., 2002). Further details are given in the Supplementary material. Briefly, 1 l of the sample was inoculated with MNV (10^6^–10^7^ copies) and solids were removed by filtration through a 10-μm hydrophilic polytetrafluoroethylene membrane and a 0.45-μm hydrophilic mixed cellulose ester (MCE) membrane. The virus concentrate (10 ml) was obtained using the adsorption-elution method. DWTP E was affected by heavy rain in August 2019 and DWTP G by a typhoon in October 2019 (turbidity of 33.7 and 219 TU-PSL, respectively). Samples measuring 0.5 l and 0.2 l from these plants, respectively, were therefore processed.

#### Viral RNA extraction

Viral RNA was extracted using the NucliSENS kit (bioMérieux, Lyon, France), following the manufacturer’s instructions. Briefly, 1 ml of viral concentrate was processed using reagents from the kit with the NucliSENS miniMAG to obtain 100 μl of RNA extract. To denature dsRNA of RVA and appropriately measure RVA concentration using real-time RT-PCR, viral RNA extracts were heated at 95°C for 5 min and immediately incubated on ice for 2 min.

### Quantification of genome copy number

Viral RNA was amplified using previously developed primers and probes for the MNV (Kitajima et al., 2010) and RVA NSP3 genes (Pang et al., 2004, 2011; Miura et al., 2018; Supplementary Table S1) using the RNA UltraSense One-Step Quantitative RT-PCR System (Thermo Fisher Scientific, Tokyo, Japan) and the LightCycler 480 System II (Roche Diagnostics, Tokyo, Japan). The reaction conditions are described in the Supplementary material. The cycle threshold (C_T_) was defined as the cycle in which the fluorescence intensity increased significantly. Undiluted and 10-fold-diluted RNA extracts were analyzed, and the absence of RT-PCR inhibition was evaluated for each sample by comparing the ΔC_T_ values against the slope of a standard curve (Miura et al., 2021). The number of genomic copies in each reaction was calculated by comparing the C_T_ value with the standard curve generated from a dilution series (10^6^–10^1^ copies/well) of plasmids containing each target region. The genome concentration in the sample was calculated based on the volume of RNA extracts analyzed and expressed as the number of copies per liter. C_T_ values < 40 were used for quantification. The limit for quantification was approximately 10^3^ copies/L of RVA in surface water samples.

Murine norovirus was added to all the samples as the whole process control virus (Haramoto et al., 2018), and the percentage of virus recovery was calculated by dividing the amount of MNV detected in the virus concentrate by the amount of MNV added to the sample. Only samples with MNV recovery efficiencies greater than 1% were considered for quantification following the criteria described in ISO 15216-1:2017 for quantification of hepatitis A virus and norovirus in food and bottled water samples (ISO, 2017). The MNV recovery efficiency was used as a quality assurance parameter and was not used for the adjustment (Kazama et al., 2017).

### RT-PCR for screening of designed primers

To screen the potential inner primers, 40 cycles of PCR were conducted twice using selected water samples (*N* = 6) from Sample Set 1. The six samples were collected between April and September, which includes both epidemic and non-epidemic seasons, and RVA concentrations were in the range of 4.1–5.5 log_10_ copies/L. Complementary DNA (cDNA) was obtained through RT using the PrimeScript RT Master Mix (Perfect Real Time, Takara Bio Inc. Kusatsu, Japan). Briefly, 100 μl of reaction mixture contained 20 μl of 5 × PrimeScript RT Master Mix, 50 μl of viral RNA extracts, and 30 μl of deionized distilled water. RT was conducted at 37°C for 15 min and 42°C for 5 min, and enzyme inactivation was conducted at 85°C for 5 s.

PCR was performed in a 25-μl reaction mixture containing 12.5 μl of the Premix Ex Taq Hot Start Version (Takara Bio Inc.), 400 nM each of forward and reverse primers, and 3 μl of cDNA. The PCR conditions were: 40 cycles at 98°C for 10 s, 55°C for 30 s, and 72°C for 30 s. PCR products were purified using the QIAquick PCR Purification Kit (Qiagen). Then, 3 μl of the purified PCR products was subjected to a second PCR cycle under the same amplification conditions with the same inner primer sets. The PCR products were visualized using 1.5% agarose gel electrophoresis.

### Nested PCR

The cDNA obtained from sample sets 1 and 2 was subjected to nested PCR assays. Briefly, the first PCR was performed in a 50-μL reaction mixture containing 25 μl of the Premix Ex Taq Hot Start Version with 500 nM each of the outer primers (Table 1) and 10 μl of cDNA, followed by a second PCR using a 50 μl of the reaction mixture consisting of 25 μl of Premix Ex Taq Hot Start Version, 500 nM each of the inner primer (Table 1), and 2 μl of the first PCR products. PCR was performed for 35 cycles at 98°C for 10 s, 50°C for 30 s, and 72°C for 30 s. The second PCR products were visualized using 1.5% agarose gel electrophoresis, and those of the required length were purified using the QIAquick PCR Purification Kit.

**Table 1:** Oligonucleotide primers specific to VP7, VP4, and VP6 RVA genes.

Name	Outer/inner Fwd./rev.	Sequence (5′ − 3′)	Location^a^	Product size
Human VP7				
H7F292_N	Outer fwd.	TGYYTRTAYTAYCCWDBHKMRG	292–313	347
H7F340_N	Outer fwd.	TGGAMRRAYWCDYTDTCNCAR	340–360	299
H7R638_N	Outer rev.	GGACAHACTTTHAYNGTRCA	619–638	−
H7F373	Inner fwd.	AARGGDTGGCCRACWRRATC	373–392	239
H7R611	Inner rev.	CCCATHGMWATCCAYTTRTTYGM	589–611	
Human VP4				
H4F793_N	Outer fwd.	TGGAARGARATGCARTATAAY	793–813	
H4R1149_N	Outer rev.	RCABWYYACDKMRTTYAAATY	1,129–1,149	357
H4R1208_N	Outer rev.	CCHCCDBTCATHAYTGGSY	1,190–1,208	416
H4F865	Inner fwd.	GGWYTRGGHTATAARTGGKCHGAA	865–888	229
H4R1093	Inner rev.	ATGCYTKHGAATCRTCCCAR	1,074–1,093	
Human VP6				
H6F648_N	Outer fwd.	GARCAYRTWGTMCAGCTHMG	648–667	462
H6R1109_N	Outer rev.	YGGRAADACTGGTCCAACTGGTAT	1,086–1,109	
H6F745	Inner fwd.	CWGAYGGMGCRACTACATGG	745–764	250
H6R994	Inner rev.	ARYGTVAGTCCWACTGTWGC	975–994	
Animal VP6				
A6F192_N	Outer fwd.	AGAAAYTGGAMWTTYRAYTTYGG	192–214	457
A6R648_N	Outer rev.	CAAAYTGYTGHRTATTWGCTGG	627–648	
A6F303	Inner fwd.	AATGTRTGYATGGATGARATRGYWMGR	303–329	272
A6R574	Inner rev.	GCRTTHADCCACATNGTNCC	555–574	

a Locations of the NCBI reference nucleotide sequence (accession no. NC011503.2 for human VP7, NC011510.2 for human VP4, and NC011509.2 for both human and animal VP6) are represented.

### DNA library preparation and NGS

To perform NGS on the RVA amplicons from Sample Set 1, unique adaptors were ligated at both the 5′ and 3′ ends of the amplicons by PCR for preparation of the DNA libraries. Further details are given in the Supplementary material. Briefly, an adapter sequence (SeqCap Adapter Kit, Roche Diagnostics, Basel, Switzerland) was ligated to the DNA fragments in the purified nested PCR products using KAPA Hyper Prep Kit (KAPA Biosystems, Inc., Wilmington, MA, United States). The ligation products were purified, and PCR (12 cycles) was performed to adjust the concentrations in the DNA libraries using KAPA HiFi Hot Start Ready Mix (KAPA Biosystems, Inc., Wilmington, MA, USA), followed by additional purification. The concentrations were measured and the quality and the expected peaks were checked using the Agilent 2,100 Bioanalyzer (Agilent Technologies, Inc., Santa Clara, CA, USA). The prepared DNA libraries were then sent to FASMAC (Atsugi, Japan), and the 229–272 bp paired-end RVA sequences were determined using the Illumina MiSeq platform (600 cycles; Illumina Inc., San Diego, CA, United States).

### Data processing and operational taxonomic unit clustering

Sequencing data processing was performed using CLC Genomics Workbench ver.11. Paired-end reads were subjected to adapter clipping and quality trimming using “Trim sequences.” Reads with Phred quality scores of less than 30 were eliminated. The OTU sequences were the nucleotide sequences that represented units for classification for sequencing reads and were determined using “*de novo* OTU clustering” with default parameters (a minimum occurrence of 2, a chimera crossover cost of 3, and a k-mer size of 6). The sequence similarity was set to a minimum of 97%, which is generally used for OTU clustering in viruses (Moniruzzaman et al., 2016; Bisseux et al., 2020). The number of OTU sequences, reads that were unmapped to OTU sequences, and the total number of reads were then extracted. The OTU sequences in each water sample, into which more than 500 reads were classified, were analyzed further if their relative abundances were greater than 1%.

Sequences similar to the determined OTU sequences were identified using megaBLAST in BLASTN (Morgulis et al., 2008). The OTU sequence the similarity of which to RVA sequences recorded in GenBank was more than 80% was regarded as an RVA sequence. When the genotype of closely-related RVA sequences (> 80% similarity) was not reported, the genotype of OTU sequence was not determined. The selected OTU sequences were indexed with the RVA genotypes and the associated hosts. To evaluate the diversity of the RVA genotypes in each water sample, the relative abundance was calculated by dividing the number of OTU sequences assigned to each genotype by the number of OTU sequences identified as RVA. The genetic diversity of RVA in DWTP E was estimated by rarefaction analysis with Analytic Rarefaction ver 2.3^2^ to check whether the genetic diversity was successfully investigated by NGS and to identify the diverse human VP7, VP4, VP6, and animal VP6 genotypes in each group.

### Phylogenetic analysis for clustering of OTU sequences

To compare the OTU nucleotide sequences from the water samples with the closely-related reference sequences from the BLASTN search, phylogenetic analysis was performed for the G, P, and I genotypes. FASTA files for each group (human VP7, VP4, VP6, and animal VP6), which included both the determined OTU and the closely-related nucleotide sequences from GenBank, were prepared for analysis. The nucleotide sequences in the FASTA files were aligned using ClustalW in MEGA X. An appropriate nucleotide substitution model was selected using the command “Find best DNA/Protein Models (ML).” The Tamura 3 parameter model (Tamura, 1992) with gamma distribution was identified for all genotypes and applied to calculate the genetic distance. Bootstrapped phylogenetic trees were constructed based on the maximum likelihood estimation with 1,000 bootstrap replications using MEGA X. The estimated phylogenetic trees were output as a Newick file, which included information about the branch length and bootstrap values and were visualized using the “ggtree” package in the R software version 3.5.0.^3^

### Accession numbers

Nucleotide sequence data were deposited in the NCBI under accession number DRA012665.

### Clinical surveillance data

The number of rotavirus gastroenteritis cases reported in the catchment upstream of DWTP E was obtained from the Infectious Diseases Weekly Report from the National Institute of Infectious Diseases, Japan (NIID, 2020). Core sentinel hospitals (approximately 500 in Japan) report the number of gastroenteritis cases caused by rotaviruses separately from those by other pathogens (e.g., noroviruses).

## Results

### Development of nested PCR assays

Based on the complete sequences of human and animal RVA VP7, VP4, and VP6 genes, eight sets of inner primers were designed and screened using selected samples (N = 6) from Sample Set 1. Amplicons were obtained for four sets of primers: H7F373/H7R611 for human VP7, H4F865/H4R1093 for human VP4, H6F745/H6R994 for human VP6, and A6F303/A6R574 for animal VP6 (Table 1). Amplicons of 229–272 bp for the screened inner primer sets were considered applicable for NGS.

To obtain amplicons with high sensitivity and specificity, outer primers were designed for the four successful inner primer sets. Six sets of outer primers that could be used for the first PCR were designed: H7F292_N/H7R638_N or H7F340_N/H7R638_N for human VP7, H4F793_N/H4R1149_N or H4F793_N/H4R1208_N for human VP4, H6F648_N/H6R1109_N for human VP6, and A6F192_N/A6R648_N for animal VP6 (Table 1). The amplicons comprising the outer primer sets were designed to be as small as possible (299–462 bp) to achieve high sensitivity.

### *Rotavirus A* concentrations in surface water samples

MNV was inoculated into the water samples from DWTP E (Sample Set 1, *N* = 12), with recovery rates ranging from 13 to 77% [mean ± standard deviation (SD), 41 ± 21%]. Real-time RT-PCR was inhibited in 5 of the 12 samples, and the C_T_ values of diluted RNA extracts were used for quantification of these five samples. The water samples from seven DWTPs (Sample Set 2, *N* = 18) resulted in MNV recovery rates ranging from 10 to 80% (mean ± SD, 36 ± 15%). Real-time RT-PCR was inhibited in 15 of the 18 samples (79%). The MNV recovery rates were therefore > 1% for both sample sets 1 and 2, indicating that no significant loss of viruses or RNA occurred during the concentration or extraction of viral RNA.

**Figure 1:**
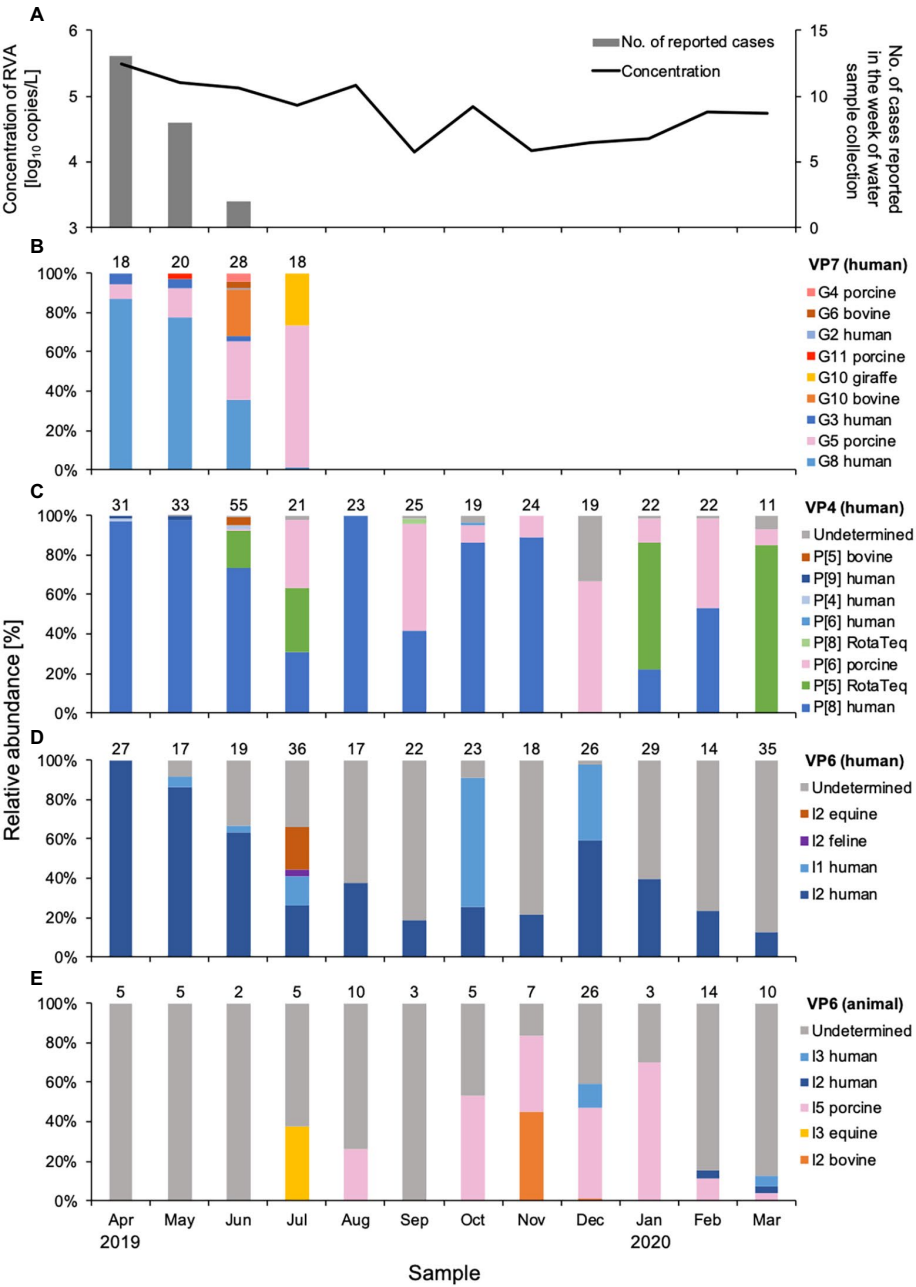
Concentration and genetic diversity of *Rotavirus A* (RVA) in water samples from DWTP E. The RVA NSP3 gene was quantitatively detected by real-time RT-PCR and plotted together with cases of rotavirus gastroenteritis reported for the week of sample collection in the prefecture upstream **(A)**. Relative abundance (%) was calculated for RVA G genotypes determined by the VP7 gene **(B)**, P genotypes determined by the VP4 gene **(C)**, and I genotypes determined by the VP6 gene amplified in human **(D)** and animal **(E)** nested PCR assays. The value above each column of relative abundance represents the number of OTUs with > 500 reads.

*Rotavirus A* concentrations were determined by quantifying the NSP3 gene, which ranged from 4.1 to 5.5 log_10_ copies/L in Sample Set 1 (Table 2, Figure 1). The concentration was high between April and August (4.9 to 5.5 log_10_ copies/L) and low between September and January, except for October (4.1 to 4.3 log_10_ copies/L). The RVA concentrations in Sample Set 2 ranged from 3.4 to 6.3 log_10_ copies/L (Table 3), which corresponds to the C_T_ values of 26.90–39.81 for undiluted RNA extracts.

**Table 2:** Sensitivity of developed nested PCR assays evaluated using Sample Set 1.^a^

			Human VP7		Human VP4		Human VP6	Animal VP6
Sample month	MNV recovery rate [%]	Conc. [log_10_ copies/L]^b^	H7F292_N/H7R638_N	H7F340_N/H7R638_N	H4F793_N/H4R1149_N	H4F793_N/H4R1208_N	H6F648_N/H6R1109_N	A6F192_N/A6R648_N
2019/4	71	5.5	−	+	+	+	+	+
5	50	5.2	−	+	+	+	+	+
6	37	5.1	−	+	+	+	+	+
7	32	4.9	−	+	−	+	+	+
8	13	5.2^c^	−	−	−	+	+	+
9	20	4.1	−	−	−	+	+	+
10	68	4.8	−	−	−	+	+	+
11	77	4.2	−	−	−	+	+	+
12	29	4.3	−	−	−	+	+	+
2020/1	31	4.3	−	−	−	+	+	+
2	32	4.8	−	−	−	+	+	+
3	30	4.7	−	−	−	+	+	+

a +/−, positive/negative for target gene.

b Concentrations determined by quantifying the NSP3 gene.

c 0.5 l was processed due to high turbidity (34 turbidity units of polystyrene latex).

**Table 3:** Sensitivity of developed nested PCR assays evaluated using Sample Set 2.^a^

				Human VP7	Human VP4		Human VP6	Animal VP6
Sample month	DWTP ID	MNV recovery rate [%]	Conc. [log_10_ copies/L]^b^	H7F340_N/H7R638_N	H4F793_N/H4R1149_N	H4F793_N/H4R1208_N	H6F648_N/H6R1109_N	A6F192_N/A6R648_N
2018/1	A	30	4.1	−	+	−	+	+
	G	43	5.3	+	+	−	+	+
	R	28	5.4	+	+	−	−	+
	U	36	6.3	+	+	−	+	+
2018/9	A	44	4.3	+	+	−	+	+
	G	33	5.0	−	+	−	+	+
	R	44	4.7	−	+	−	+	+
	U	33	5.7	−	+	−	+	+
2018/10	O	47	4.7	+	+	−	−	+
2019/1	G	34	5.0	−	+	−	+	+
2019/2	A	45	4.5	−	+	−	+	+
2019/10	C	20	4.6	+	+	−	+	+
	G	29	3.5^c^	−	−	−	−	−
2020/1	A	80	4.1	+	+	−	+	+
	C	33	4.5	+	+	+	+	+
	D	32	3.7	−	+	+	+	+
2020/10	A	21	3.4	+	−	+	+	+
	U	10	4.5	−	−	−	+	+

a DWTP, drinking water treatment plant; +/−, positive/negative for target gene.

b Concentrations determined by quantifying the NSP3 gene.

c 0.2 l processed due to high turbidity (219 turbidity units of polystyrene latex).

### Sensitivity of the developed nested PCR assays

Six sets of nested PCR assays were tested for sensitivity using Sample Set 1, with amplicons obtained from five (Table 2). Hereafter, each assay is specified by the names of outer primer sets. No amplicons for the human VP7 gene were obtained using the outer primer sets H7F292_N/H7R638_N; however, the amplicons were found using H7F340_N/H7R638_N in samples collected between April and July when RVA concentrations were high. Amplicons for the human VP4 gene were obtained only from high-concentration samples collected between April and June using the outer primers H4F793_N/H4R1149_N, whereas amplicons for the VP4 genes were obtained from all samples using H4F793_N/H4R1208_N. The two assays for human and animal VP6 genes could produce amplicons in all samples. The sensitivity of the five nested PCR assays was therefore considered sufficient for water samples affected by both human and animal feces throughout the year.

To further evaluate the sensitivity of the five assays where the amplicons were obtained, Sample Set 2 (N = 18) from seven DWTPs with wider RVA concentrations was employed (Table 3). Amplicons were obtained from 17 of the 18 samples by at least one of the assays. No amplicons were obtained from the sample with the second lowest concentration (3.5 log_10_ copies/L), which was affected by a typhoon (turbidity, 219 TU-PSL) before collection from DWTP G in October 2019, inhibiting real-time RT-PCR [based on the C_T_ of undiluted and 10-fold diluted RNA extracts were 31.37 and 32.16, respectively (ΔC_T_ = 0.79) for MNV detection; C_T_ of undiluted RNA extract was 39.81 for NSP3 gene detection]. However, amplicons of human VP7, VP4, and VP6, and animal VP6 genes were successfully obtained from the sample with the lowest concentration (3.4 log_10_ copies/L). The nine samples in which the VP7 gene was amplified were also positive for the VP4 and VP6 genes. In contrast to Sample Set 1, the outer primers H4F793_N/H4R1149_N were found to obtain more amplicons than H4F793_N/H4R1208_N. The nested PCR assays for the animal VP6 gene could obtain amplicons from all samples, except the one that was affected by the typhoon. These results suggested that the five nested PCR assays have a potential to obtain amplicons from surface water samples as long as the NSP3 gene is detected by using real-time RT-PCR.

### Amplicon-based NGS

A total of 43 amplicon samples were obtained for human VP7, VP4, and VP6, and animal VP6 genes from Sample Set 1 and subjected to NGS. After filtering the nucleotide sequences (denoising and chimera removal), a total of 2,635,931 sequence reads were obtained as RVA reads, with a mean of 61,300 per amplicon sample. The mean number of OTUs with more than 500 reads per amplicon sample was 17.8 (range, 2–37, Supplementary Table S2). G, P, or I genotype was determined for each OTU sequence, and the relative abundance was calculated for each sample (Figure 1). Based on the megaBLAST analysis, we confirmed that there was no cross-reaction with non-rotavirus sequences. Rarefaction curves for each amplicon sample (Supplementary Figure S1) and prevalent genotype (Supplementary Figure S2) plateaued, indicating that the genetic diversity was comprehensively ascertained in the amplicon-based NGS.

#### G genotypes determined by VP7 sequence analysis

Nine G genotypes (maximum seven genotypes per sample) were detected in water samples collected between April and July 2019 (Figure 1B). The human G8 genotype was predominant (relative abundance, 36–87%) in samples from April and June, the rotavirus season in Japan. The relative abundance of the G8 genotype decreased as reports of rotavirus infection decreased. The human G3 genotype was detected with a relative abundance of 2.4–5.8% during the epidemic season, while small amounts (0.7%) of the G2 genotype were found only in June. Although the assay was based on human VP7 sequences, porcine G5, G11, and G4, and bovine G10 and G6 genotypes were also detected. Porcine G5 was detected each month between April and July, and its relative abundance increased as human G8 decreased. Bovine G10 and G6 genotypes were detected sporadically in June.

**Figure 2:**
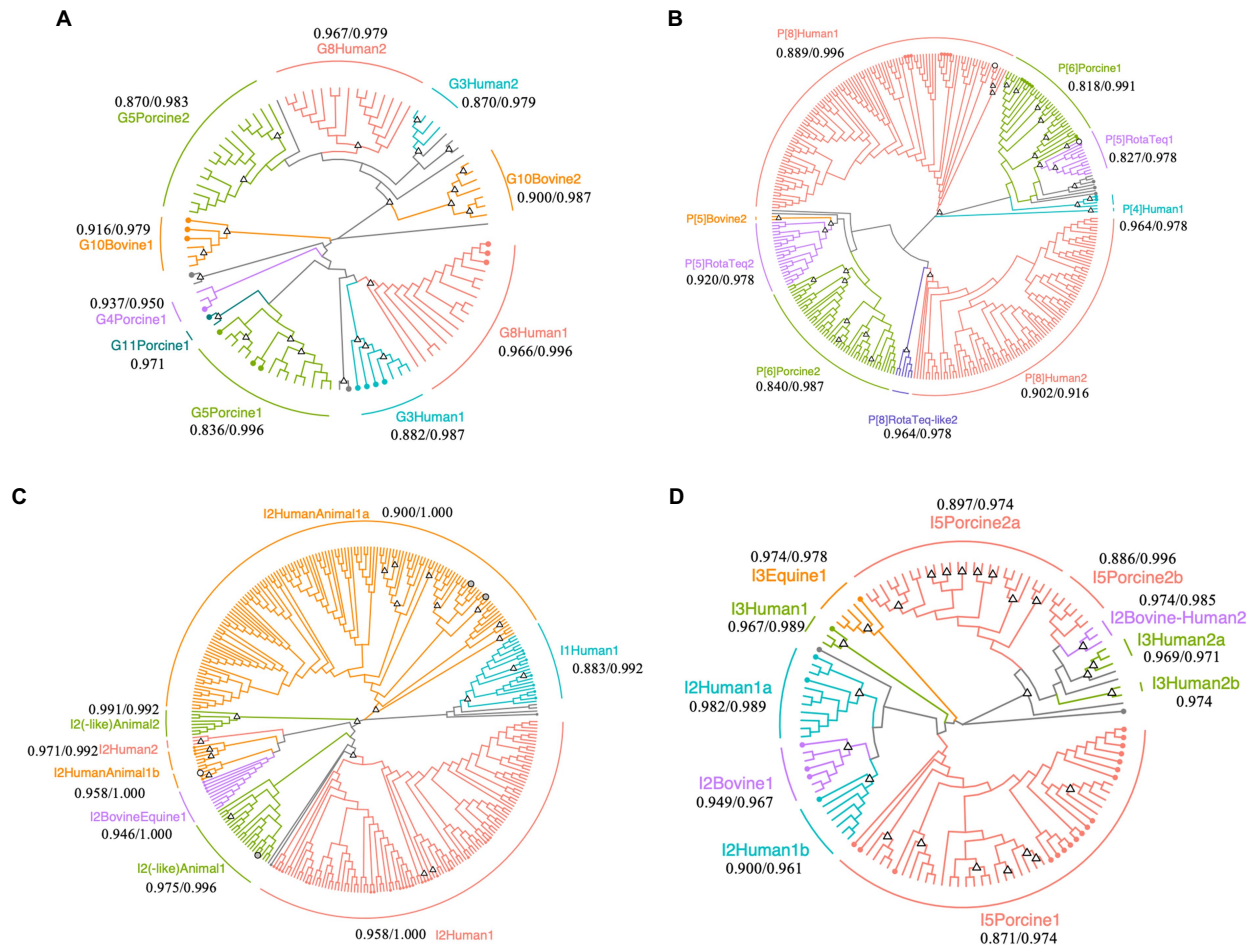
Phylogenetic trees of *Rotavirus A* strains detected in surface water samples collected at DWTP E. Trees were built for partial regions of the VP7 **(A)**, VP4 **(B)**, and VP6 genes **(C,D)** obtained using human and animal nested PCR assays with maximum likelihood and bootstrapped with 1,000 repetitions. The sequence lengths of VP7, VP4, and human and animal VP6 were 242, 230, 239, and 274  bp, respectively. Circles with branch colors represent reference sequences that are closely related to OTU sequences. White and gray circles represent reference sequences for RotaTeq^®^ and closely-related OTU sequences. The information of reference sequences is provided in Supplementary Table S3 in Supplementary material. Triangles at the nodes represent bootstrap rates of >0.8. The name of each sub-cluster consists of the genotype, followed by host or RotaTeq^®^, 1 (cluster with reference sequences) or 2 (cluster with only OTU sequences), and a or b for more than two sub-clusters. The values represent the minimum/maximum sequence similarity within each sub-cluster.

Phylogenetic analysis of the VP7 sequences revealed that human G8 and G3, porcine G5, and bovine G10 (including giraffe G10) OTU sequences are clustered in two sub-clusters (Figure 2A). One G8 OTU sub-cluster contained sequences that were isolated from the stool samples of gastroenteritis patients in Japan (accession No., LC477362.1; isolated in 2017), the Czech Republic (accession No., MN401299.1; isolated in 2017), and South Korea (accession No., MN058762.1; isolated in 2017) (maximum and minimum sequence similarity within the sub-cluster: 0.966 and 0.996), while the other sub-cluster comprised only OTU sequences (maximum and minimum sequence similarity: 0.979 and 0.967). Similarly, the G3 OTU sequences in one sub-cluster were closely related to those isolated from gastroenteritis patients in Japan (accession No., LC340021.1 and AB919147.1; isolated in 2014 and 2011), Russia (accession No., KF018794.2; isolated in 2007), and Vietnam (accession No., LC433666.1; isolated in 2016), while the remaining OTU sequences formed another sub-cluster (maximum and minimum sequence similarity: 0.979 and 0.870).

#### P genotypes determined by VP4 sequence analysis

A total of eight P genotypes (a maximum of five genotypes per sample) were identified in the amplicons obtained by the two nested PCR assays for VP4 genes. The human P[8] genotype was detected throughout the year, except for December 2019 and March 2020, and was the predominant genotype (relative abundance, 22–100%) in most months (Figure 1C). Human P[4] and P[9] genotypes were detected during the epidemic season, amplified by the outer primers H4F793_N/H4R1149_N, and P[5] and P[8] RotaTeq® sequences were observed sporadically in June, July, and September 2019, and January and March 2020. Rotarix® sequences were not detected in any sample. Porcine and bovine genotypes were also detected. The porcine P[6] genotype was detected between June and March, with a relative abundance of 0.6–67%. The bovine P[5] genotype was found only in June, with a small abundance (3.7%), at the same time as bovine G10 and G6 genotypes were detected.

Operational taxonomic unit sequences belonging to the human P[8] genotype formed two sub-clusters in the phylogenetic tree, with some sequences closely related to those isolated from gastroenteritis patients in Singapore, Thailand, South Korea, Vietnam, Dominican Republic, United States, and Japan (MG996086.1, LC514473.1, MN058759.1, LC491560.1, MG652355.1, MF168111.1, and LC172539.1; Figure 2B). The sequence similarities within the human P[8] sub-cluster, which included reference sequences, were 0.996 and 0.889 at maximum and minimum, respectively. Another human P[8] sub-cluster composed of only OTU sequences also showed high sequence similarity (0.916 and 0.902 at maximum and minimum). The P[8] RotaTeq®-like OTU sequences formed another cluster (maximum and minimum sequence similarity: 0.978 and 0.964), and P[5] RotaTeq®-like OTU sequences formed two sub-clusters, one of which included a reference sequence (MF469343.1). The maximum/minimum sequence similarities of two P[5] RotaTeq sub-clusters were 0.978/0.827 (a sub-cluster including reference sequences) and 0.978/0.920 (another composed of only OTU sequences). Porcine P[6] OTU sequences in one of these sub-clusters were closely related to sequences isolated from pigs in the USA, Taiwan, Japan, and China (KR052749.1, MK227950.1, AB924098.1, and MG570048.1), human stool in Thailand (KY748311.1), and wild boar in Japan (AB573872.1; maximum and minimum sequence similarity: 0.991 and 0.818), while the other OTU sequences formed another sub-cluster (maximum and minimum sequence similarity: 0.987 and 0.840).

#### I genotypes determined by VP6 sequence analysis

Analysis of the VP6 sequences obtained with the human VP6 nested PCR assay resulted in identification of the human I2 and I1 genotypes (Figure 1D). The human I2 genotype was predominant during the epidemic season, when it was detected every month with a relative abundance of 13–100%. The human I1 genotype was sporadic and abundant in October and December 2019 (66 and 38%, respectively) compared to the I2 genotype. Feline I2 and equine I2 genotypes were detected with relative abundances of 3.2 and 21%, respectively, in July 2019. The genotypes of some OTU sequences were not specified because no closely-related sequences were recorded in GenBank.

Of the VP6 sequences that were obtained by the animal VP6 nested PCR assay, the porcine I5 genotype detected between August 2019 and March 2020 was predominant (Figure 1E). The equine I3 genotype showed a relative abundance of 38% in July when the equine I2 genotype was present. Bovine I2 genotype was detected in November and December with relative abundances of 45 and 1.5%, respectively, whereas no bovine genotypes were detected in June when G10, G6, and P[5] were found. All of the OTU sequences obtained in April, May, June, and September were undetermined. Relatively low abundances of human I2 and I3 genotypes were detected between December and March (3.1–12%).

Phylogenetic analysis of the human VP6 nested PCR amplicons revealed diverse human I2 OTU sequences (Figure 2C). RotaTeq^®^ is a pentavalent rotavirus vaccine that contains five human-bovine reassortant strains, in which the VP6 gene is classified as the bovine I2 genotype (Matthijnssens et al., 2010). I2 OTU sequences were closely related to a reference sequence of the reassortant vaccine strain G4 (KC215501.1), clustered into a sub-cluster of human and animal I2 genotypes (maximum and minimum sequence similarities: 1.000 and 0.958).

Porcine I5 OTU sequences formed three sub-clusters in the phylogenetic tree of the animal VP6 sequences (Figure 2D). One sub-cluster contained reference sequences from pigs in Spain, China, Slovakia, Taiwan, Japan, South Africa, Belgium, and the Czech Republic (MH238295.1, MH238299.1, MK936420.1, MK936421.1, EU372799.1, MK410285.1, MN203570.1, MK597975.1, MK597964.1, KF303566.1, MF139477.1, KU739976.1, AB924088.1, AB924099.1, KP753126.1, KM820727.1, and KU887650.1; maximum and minimum sequence similarities: 0.974 and 0.871), while the other sub-clusters comprised only OTU sequences (maximum and minimum sequence similarities: 0.974 and 0.897). Equine I3 OTU sequences were closely related to a reference sequence from a horse in Argentina (JX036369.1; maximum and minimum sequence similarities: 0.978 and 0.974) and the bovine I2 OTU sequences were closely related to reference sequences from cows in Japan (LC553621.1, AB853894.1, and AB573082.1; maximum and minimum sequence similarities: 0.967 and 0.949).

## Discussion

Surface water contains both human and animal RVA, and a method for analyzing low concentrations of the diverse RVA genotypes is required to evaluate the potential health risks associated with water usage. In this study, five nested PCR assays for human RVA VP7, VP4, and VP6 genes and the animal RVA VP6 gene were successfully developed. The amplicons derived from water samples obtained each month (Sample Set 1) were subjected to NGS and the diverse RVA genotypes and OTU sequences were obtained. The results indicated that the abundance of the identified RVA genotypes varied over the study period.

An amplicon-based NGS approach was applied to water samples collected at DWTPs to analyze the diverse RVA genotypes present. RVA concentrations are generally lower in drinking water sources than sewage, rendering it difficult to obtain appropriate amplicons for genotyping. The five nested PCR assays developed succeeded in amplifying parts of the human VP7, VP4, and VP6 genes and animal VP6 genes from water samples with concentrations as low as 3.4 log_10_ copies/L (C_T_, 37.67). Nine G (human G8, G3, G2, porcine G5, G11, G4, bovine G10, G6, and giraffe G10), eight P (human P[8], P[6], P[4], P[9], RotaTeq P[5], P[8], porcine P[6], bovine P[5]), and eight I (human I1, I2, I3, porcine I5, bovine I2, feline I2, equine I2, I3) genotypes were identified. Although obtaining a whole sequence of an RNA segment is informative, as reference sequences of the same genotype formed a distinct cluster in the phylogenetic trees, the sequence lengths of 200–300 bp can be applicable to determine RVA G, P, and I genotypes. The lengths were suitable for our purposes of obtaining amplicons from environmental water samples and performing NGS. Although both human and animal sequences were unexpectedly amplified partly because we designed degenerate primers, this was advantageous for our purpose to understand diverse RVA genotypes detected in environmental water. An amplicon-based NGS approach can also be applicable to other RVA genome segments; however, the number of recorded sequences would be smaller compared to VP7, VP4, and VP6 genes which have been used for conventional genotyping. As for the sewage samples where RVA concentrations were generally higher, the full length of VP7 (1,063 bp) and VP4 (2,359 bp) genes was obtained and 9 G and 13 P genotypes were detected from eight sewage samples in China by NGS (Du et al., 2021). Adriaenssens et al. (2018) used a viromic (viral metagenomics) approach to investigate human pathogenic RNA viruses in sewage and detected RVA genome fragments from the G10, G8, P[1], P[14] P[41], and I2 genotypes. To our knowledge, RVA genome fragments have not been detected in drinking water sources by using viromic approaches. Viromic approaches have a potential to detect novel viruses or genotypes (Nieuwenhuijse and Koopmans, 2017; Adriaenssens et al., 2018), while the amplicon-based approach can detect viruses in low concentrations and observe the genetic diversity in depth, as demonstrated in this study.

Human G8, P[8], and I2 genotypes, which were predominant in water samples from April 2019, decreased in relative abundance over the next few months, alongside a decrease in the number of cases reported (Figure 1). The human G3 genotype detected between April and June was at lower relative abundance than the G8 genotype. The G8P[8] genotype, which was first reported in Hokkaido in 2014 (Kondo et al., 2017), and the G8 genotype were predominant in patients with gastroenteritis during the epidemic season between 2018 and 2019 in Japan (Tsugawa et al., 2021). An equine-like G3P[4] strain was first isolated in Sendai in 2013 (Malasao et al., 2015) and equine-like G3P[8] strains have been identified in gastroenteritis patients since 2015 (Kikuchi et al., 2018; Komoto et al., 2018). The G8P[8], equine-like G3P[4], and equine-like G3P[8] strains have the I2 genotype (Malasao et al., 2015; Tsugawa et al., 2021), which is consistent with our observation that I2 was the predominant I genotype together with G8 and P[8] in the water samples. In 2018 and 2019, on average 1–3 cases of rotavirus-associated gastroenteritis were weekly reported in February or March in the catchment area (Supplementary Table S4; NIID, 2020), and the concentration of RVA in the surface water increased in 2019 (Supplementary Figure S3). However, no cases were reported in February and March 2020, and RVA concentrations remained stable (Figure 1). This may be due to the closure of schools from March to May 2020, when most schools and nurseries were closed in the coronavirus infectious disease 2019 pandemic. This may explain why the RotaTeq^®^ P[5] genotype predominated water samples in March 2020. Thus, the RVA genotypes in the river water samples can be assumed to reflect the genotypes circulating in human populations, as demonstrated for noroviruses in sewage (Kazama et al., 2017).

In phylogenetic trees, some OTU sequences of the same genotype were classified into different sub-clusters (Figure 2). The sequence similarities between the sub-clusters which were classified as the same genotype were less than 0.7, which suggests that there are not a small number of non-synonymous mutations in one sub-cluster and such nontrivial mutations lead to the formation of multiple sub-clusters. Most OTU sequences of the VP4 gene were classified as P[8], which is composed of several clades, but the bootstrap probability did not exceed 0.8. These results indicate that the human P[8] genotype has high genetic diversity due to a high evolutionary rate, and many variants exist in drinking water sources. In addition to the VP4 gene, OTU sequences of the human I2 genotype within the same sub-clusters were closely related. As high genetic diversity is advantageous for viral spread (Bordería et al., 2015; Kadoya et al., 2020), it is important that the genetic diversity of RVA is appropriately decreased during the water treatment processes.

RotaTeq^®^ P[5] and P[8] genotypes were detected regardless of season, and OTU sequences similar to the RotaTeq^®^ reference sequences, which had not been detected in surface water, were identified in this study. Less RotaTeq^®^ (7.8–11%) vaccinations were given in the catchment area between April 2019 and March 2020 compared to Rotarix® (66–94%), as estimated by the number of each vaccine provided by GlaxoSmithKline K.K. and MSD K.K. and the number of live births. Our observation that only RotaTeq^®^ OTU sequences were identified in the surface water samples could be explained by the different use of Rotarix^®^ and RotaTeq^®^, as pointed by Ito et al. (2021). Inoculation with Rotarix® is conducted twice before 24 weeks and RotaTeq^®^ three times before 32 weeks. Infants generally begin weaning at 20 to 24 weeks in Japan, and their feces are incorporated into the sewage. Therefore, more RotaTeq^®^ is likely to reach the environmental waters.

The porcine G5 genotype was more frequently detected than the G4 and G11 genotypes, with P[6] the only porcine P genotype identified. Pigs can become infected with several different strains of RVA and the G5 genotype was detected most frequently, followed by G11, G2, G9, and G4, at a farm in Japan (Miyazaki et al., 2012). The predominant RVA genotypes in each developmental stage change annually, with the G4P[13], G5P[6], and G9P[6] genotypes prevalent from 2006 to 2008 (Miyazaki et al., 2013). Although the predominant RVA genotypes in swine populations inhabiting the catchment area are unknown, the G5 and P[6] genotypes are likely to have dominated during the study period. Imagawa et al. (2020) reported that G5 was the most frequently detected genotype in river water samples from the Philippines during 2012–2013 and G5P[6] and G4P[6] were identified in clinical samples, which may point to reassortment between human and porcine RVA. Porcine G4 and G5 serotypes have been found to be closely related to RVA strains circulating in humans, suggesting that swine play a crucial role as reservoir and generator of newly adapted and emerging strains in humans (Dhama et al., 2009).

Although human G8, P[8], and I2 genotypes were predominant in the water samples collected between April and June, other host species identified by the detected genotypes did not always match within a water sample. This was because animal genotypes were detected as well as human genotypes and the amplification efficiencies may be different from strain to strain. As the nested PCR assays developed in this study may detect both human and animal RVA strains, the amplicons need to be sequenced. The two outer primer sets for VP4 (H4F793_N/H4R1149_N; H4F793_N/H4R1208_N) presented different results for sample sets 1 and 2, and human P[4] and P[9] genotypes were detected only by H4F793_N/H4R1149_N. As it has no known cause, the detection sensitivities and genotypes detected by the two outer primer sets require further evaluation. To increase the chance of obtaining amplicons, we recommend to use the two outer primer sets for the VP4 gene. These are the limitations in use of developed assays.

In this study, the OTU sequence, the similarity of which to RVA sequences recorded in GenBank was more than 80%, was regarded as an RVA sequence. We confirmed there were no non-rotavirus sequences based on the megaBLAST analysis of obtained OTU sequences. As for the undetermined RVA sequences, these sequences were certainly rotavirus ones but the genotype and associated host were not reported for the closely-related RVA sequences found in GenBank. If new animal source of rotavirus, new genotype, or variants are found and their sequences are registered properly, the genotype and host of currently undetermined RVA sequences can be revealed.

The genotype distribution of RVA in the epidemic season varies in different areas (Tsugawa et al., 2021). Therefore, continuous investigation of the RVA strains that are in circulation is necessary to elucidate the predominant genotype and detect the emergence of novel genotypes. The established primer sets successfully captured various RVA genotypes in the water and could be used to monitor changes in the RVA genotypes present. Genotypes with higher relative abundance are likely associated with RVA cases at the time of monitoring, meaning that monitoring is useful for alerting RVA outbreaks. Changes in the genotype distribution after mass vaccination in developing countries with limited clinical surveillance systems can also be evaluated using our approach. The reassortment of human RVA and RotaTeq® strains or human and animal RVA strains has been reported previously (Bucardo et al., 2012). As our primer sets detect both human and animal RVA, our approach may also be applicable to the investigation of animal RVA genotypes in the environment. However, it remains unknown which genotypes our primer sets can detect and whether specific genotypes are more prone to be captured by the primer sets.

In conclusion, nested PCR assays were newly developed to amplify partial regions of the VP7, VP4, and VP6 genes of RVA for amplicon-based NGS to investigate the RVA genotypes in water samples. The assays demonstrated sufficient sensitivity to obtain amplicons from drinking water sources with concentrations as low as 3.4 log_10_ copies/L, and a large diversity of RVA genotypes was identified. More observations from around the world would be useful to better understand the genetic diversity of both human and animal RVA in drinking water sources, which will be considered in risk assessment.

## Data availability statement

The datasets presented in this study can be found in online repositories. The names of the repository/repositories and accession number(s) can be found in the article/Supplementary material.

## Author contributions

TM developed the concept and drafted the first manuscript. TM, S-sK, and HT performed the primer design, water sample processing, RT-PCR, and bioinformatics analyses. S-sK, HT, DS, and MA reviewed and edited the manuscript. All authors contributed to the article and approved the submitted version.

## Funding

This work was supported by the Ministry of Health, Labour, and Welfare, Japan, through a Health and Labour Sciences Research Grant (19LA1005 and 22LA1007) and by the Japan Society for the Promotion of Science through KAKENHI (JP19K04680 and JP20H04359).

## Conflict of interest

The authors declare that the research was conducted in the absence of any commercial or financial relationships that could be construed as a potential conflict of interest.

## Publisher’s note

All claims expressed in this article are solely those of the authors and do not necessarily represent those of their affiliated organizations, or those of the publisher, the editors and the reviewers. Any product that may be evaluated in this article, or claim that may be made by its manufacturer, is not guaranteed or endorsed by the publisher.

## Footnotes

^1^ https://www.ncbi.nlm.nih.gov/nucleotide/

^2^ http://strata.uga.edu/software/

^3^ https://www.r-project.org/
