# Supplementary material for: Temporal variations of human and animal Rotavirus A genotypes in surface water used for drinking water production
Source: Front Microbiol. 2022 Aug 9;13:912147. doi: 10.3389/fmicb.2022.912147 (PMC9395708; doi:10.3389/fmicb.2022.912147)
Supplement: Supplementary file 1 [file Data_Sheet_1.docx]

Supplementary Material

# Virus concentration procedures

Water samples (1 L) were inoculated with MNV (10^6^–10^7^ copies) and filtered first through a 10-μm hydrophilic polytetrafluoroethylene membrane (90-mm diameter; JCWP09025, Merck, Tokyo, Japan). The filtrate was recovered in a sterile suction bottle and was then filtered through a 0.45-μm hydrophilic mixed cellulose esters (MCE) membrane (90-mm diameter; HAWP09000, Merck, Tokyo, Japan). The filtrate was recovered in another sterile suction bottle and magnesium chloride hexahydrate was added at a final concentration of 25 mM. The solution was filtered through a 0.45-μm MCE membrane and the membrane was rinsed with 200 mL of 0.5 mM H_2_SO_4_ (pH 3.0). Subsequently, 10 mL of 1 mM NaOH (pH 11.0) was filtered through the membrane to elute viruses. The virus concentrate (10 mL filtrate) was recovered to a 50-mL tube containing 100 μL of 50 mM H_2_SO_4_ and 100 μL of 100×Tris-EDTA (TE) buffer for neutralization.

# Reaction conditions of real-time RT-PCR

The 25 µL reaction mixture comprised 5 μL of 5× reaction mix, 1.25 μL enzyme mix, 500 nM forward primer, 900 nM RT and reverse primer, 125 nM probe, and 5 μL of viral RNA extracts. The RT-PCR conditions were as follows: 55°C for 15 min and 95°C for 15 s, followed by 45 cycles of 95°C for 15 s, 60°C for 30 s, and 65°C for 30 s.

# DNA library preparation procedures

DNA concentrations of purified nested PCR products were adjusted to 2.5 ng/50 µL according to the manufacturer’s protocol of KAPA Hyper Pre Kit (Kapa Biosystems, Inc., Wilmington, MA, USA). Both the 5’ and 3’ ends of the DNA fragments were treated with End-repair & A-Tailing enzyme of a KAPA Hyper Prep Kit. Ligation was performed using a SeqCap Adapter Kit (Roche Diagnostics) and a Kapa Hyper Prep Kit with 750 nM adapters following the manufacturer’s instruction. The ligation products were purified using an AMPureXP (Beckman Coulter, Tokyo, Japan). PCR (12 cycles) was performed to adjust concentrations of DNA libraries using a KAPA HiFi HS ReadyMix (Kapa Biosystems, Inc., Wobrun, MA, USA) followed by two times purification using an AMPureXP (Beckman Coulter). The concentrations of DNA library were measured using a Qubit 4 Fluorometer (Thermo Fisher Scientific, MA, USA), and the quality of prepared DNA libraries and the expected peaks were checked using an Agilent 2100 Bioanalyzer (Agilent Technologies, Inc., Santa Clara, CA, USA)

# Supplementary Tables

**Table S1**. Oligonucleotide sequences of primers and probes used in real-time RT-PCR.

| Primer or probe | Sequence (5’ – 3’) | Reference |
| --- | --- | --- |
| Murine norovirus | |  |
| MNV-S | CCGCAGGAACGCTCAGCAG | Kitajima et al. 2010 |
| MNV-AS | GGYTGAATGGGGACGGCCTG |  |
| MNV-TP | FAM-ATGAGTGATGGCGCA-MGB-NFQ |  |
| *Rotavirus A* | |  |
| NVP3-F | ACCATCTACACATGACCCTC | Pang et al. 2004 |
| Primer-F2 | ACCATCTTCACGTAACCCTC | Pang et al. 2011 |
| NVP3-R | GGTCACATAACGCCCC | Pang et al. 2004 |
| RVA-MGB | FAM-TGAGCACAATAGTTAAAAGC-MGB-NFQ | Miura et al. 2018 |

FAM, 6-carboxyfluorescein; TAMRA, 5-Carboxytetramethylrhodamine; MGB, minor groove binder; NFQ, nonfluorescent quencher

**References**

Kitajima, M., Oka, T., Takagi, H., Tohya, Y., Katayama, H., Takeda, N., et al. (2010). Development and application of a broadly reactive real-time reverse transcription-PCR assay for detection of murine noroviruses. *Journal of Virological Methods* 169(2), 269-273. doi: 10.1016/j.jviromet.2010.07.018.

Pang, X., Cao, M., Zhang, M., and Lee, B. (2011). Increased sensitivity for various rotavirus genotypes in stool specimens by amending three mismatched nucleotides in the forward primer of a real-time RT-PCR assay. *Journal of Virological Methods* 172(1–2), 85-87. doi: 10.1016/j.jviromet.2010.12.013.

Pang, X.L., Lee, B., Boroumand, N., Leblanc, B., Preiksaitis, J.K., and Yu Ip, C.C. (2004). Increased detection of rotavirus using a real time reverse transcription-polymerase chain reaction (RT-PCR) assay in stool specimens from children with diarrhea. *Journal of Medical Virology* 72(3), 496-501. doi: 10.1002/jmv.20009.

Miura, T., Schaeffer, J., Le Saux, J.-C., Le Mehaute, P., and Le Guyader, F.S. (2018). Virus type-specific removal in a full-scale membrane bioreactor treatment process. *Food and Environmental Virology* 10(2), 176-186. doi: 10.1007/s12560-017-9330-4.

**Table S2**. The number of sequence reads and OTUs

| Sample month | Total reads | Reads filtered out | % filtered out | RVA reads | No. of OTUs | No. of OTUs  (>500 reads) |
| --- | --- | --- | --- | --- | --- | --- |
|  |  |  |  |  |  |  |
| **VP7 (human, H7F340_N/H7R638_N)** | | |  |  |  |  |
| 2019/4 | 206,360 | 101,932 | 49.4 | 104,428 | 68 | 18 |
| 2019/5 | 185,251 | 93,621 | 50.5 | 91,630 | 54 | 20 |
| 2019/6 | 212,769 | 120,341 | 56.6 | 92,428 | 88 | 28 |
| 2019/7 | 166,511 | 74,521 | 44.8 | 91,990 | 43 | 18 |
| **Total** | **770,891** | **390,415** |  | **380,476** | **253** | **84** |
|  |  |  |  |  |  |  |
| **VP4 (human, H4F796_N/H4R1152_N)** | | |  |  |  |  |
| 2019/4 | 115,802 | 66,116 | 57.1 | 49,686 | 47 | 12 |
| 2019/5 | 140,594 | 65,883 | 46.9 | 74,711 | 32 | 15 |
| 2019/6 | 119,163 | 76,479 | 64.2 | 42,684 | 64 | 18 |
| 2019/7 | 132,563 | 83,041 | 62.6 | 49,522 | 53 | 21 |
| 2019/8 | 113,868 | 42,049 | 36.9 | 71,819 | 33 | 23 |
| 2019/9 | 133,672 | 80,734 | 60.4 | 52,938 | 51 | 25 |
| 2019/10 | 121,936 | 57,239 | 46.9 | 64,697 | 46 | 19 |
| 2019/11 | 129,679 | 43,933 | 33.9 | 85,746 | 35 | 24 |
| 2019/12 | 102,934 | 55,570 | 54.0 | 47,364 | 45 | 19 |
| 2020/1 | 151,376 | 67,906 | 44.9 | 83,470 | 48 | 22 |
| 2020/2 | 159,187 | 68,764 | 43.2 | 90,423 | 36 | 22 |
| 2020/3 | 83,036 | 38,297 | 46.1 | 44,739 | 33 | 11 |
| **Total** | **1,503,810** | **746,011** |  | **757,799** | **523** | **231** |
|  |  |  |  |  |  |  |
| **VP4 (human, H4F796_N/H4R1211_N)** | | |  |  |  |  |
| 2019/4 | 166,658 | 85,899 | 51.5 | 80,759 | 65 | 19 |
| 2019/5 | 132,563 | 59,873 | 45.2 | 72,690 | 41 | 18 |
| 2019/6 | 164,111 | 83,440 | 50.8 | 80,671 | 74 | 37 |
| **Total** | **463,332** | **229,212** |  | **234,120** | **180** | **74** |
|  |  |  |  |  |  |  |
| **VP6 (human, H6F648_N/H6R1109_N)** | | |  |  |  |  |
| 2019/4 | 184,897 | 93,922 | 50.8 | 90,975 | 69 | 27 |
| 2019/5 | 144,208 | 63,618 | 44.1 | 80,590 | 40 | 17 |
| 2019/6 | 123,736 | 66,805 | 54.0 | 56,931 | 49 | 19 |
| 2019/7 | 177,274 | 90,092 | 50.8 | 87,182 | 71 | 36 |
| 2019/8 | 132,096 | 63,218 | 47.9 | 68,878 | 29 | 17 |
| 2019/9 | 133,663 | 58,527 | 43.8 | 75,136 | 39 | 22 |
| 2019/10 | 234,267 | 74,379 | 31.7 | 159,888 | 41 | 23 |
| 2019/11 | 124,170 | 37,432 | 30.1 | 86,738 | 28 | 18 |
| 2019/12 | 139,935 | 61,775 | 44.1 | 78,160 | 42 | 26 |
| 2020/1 | 150,482 | 79,257 | 52.7 | 71,225 | 70 | 29 |
| 2020/2 | 86,515 | 35,397 | 40.9 | 51,118 | 26 | 14 |
| 2020/3 | 235,987 | 117,203 | 49.7 | 118,784 | 59 | 35 |
| **Total** | **1,867,230** | **841,625** |  | **1,025,605** | **563** | **283** |
|  |  |  |  |  |  |  |
| **VP6 (animal, A6F192_N/A6R648_N)** | | |  |  |  |  |
| 2019/4 | 126,510 | 114,217 | 90.3 | 12,293 | 64 | 5 |
| 2019/5 | 115,126 | 100,697 | 87.5 | 14,429 | 41 | 5 |
| 2019/6 | 119,447 | 113,429 | 95.0 | 6,018 | 70 | 2 |
| 2019/7 | 129,521 | 120,569 | 93.1 | 8,952 | 58 | 5 |
| 2019/8 | 145,945 | 127,590 | 87.4 | 18,355 | 36 | 10 |
| 2019/9 | 132,744 | 121,231 | 91.3 | 11,513 | 43 | 3 |
| 2019/10 | 147,390 | 135,296 | 91.8 | 12,094 | 43 | 5 |
| 2019/11 | 124,244 | 99,538 | 80.1 | 24,706 | 29 | 7 |
| 2019/12 | 316,858 | 254,209 | 80.2 | 62,649 | 65 | 26 |
| 2020/1 | 126,625 | 121,130 | 95.7 | 5,495 | 59 | 3 |
| 2020/2 | 203,522 | 173,544 | 85.3 | 29,978 | 59 | 14 |
| 2020/3 | 208,319 | 176,870 | 84.9 | 31,449 | 61 | 10 |
| **Total** | **1,896,251** | **1,658,320** |  | **237,931** | **628** | **95** |
|  |  |  |  |  |  |  |
| **Ground total** | **6,501,514** | **3,865,583** |  | **2,635,931** | **2147** | **767** |
|  |  |  |  |  |  |  |
| Mean | 151,198.0 |  |  | 61,300.7 | 49.9 | 17.8 |
| Min | 83,036 |  |  | 5,495 | 26 | 2 |
| Max | 316,858 |  |  | 159,888 | 88 | 37 |

**Table S3**. Information of reference sequences used for phylogenetic analysis (NCBI accession number, genotype, host, year, and country or region in which the strain was isolated).

| Accession No. | Genotype | Host | Year | Country or region |
| --- | --- | --- | --- | --- |
| **VP7 (human)** |  |  |  |  |
| LC477359.1 | G2 | human | 2017 | Japan |
| AB919147.1 | G3 | human | 2011 | Japan |
| LC433666.1 | G3 | human | 2016 | Vietnam |
| KF018794.2 | G3 | human | 2007 | Russia |
| LC340021.1 | G3 | human | 2014 | Japan |
| MN401299.1 | G8 | human | 2017 | Czech Republic |
| MN058762.1 | G8 | human | 2017 | South Korea |
| LC477362.1 | G8 | human | 2017 | Japan |
| KP836291.1 | G3 | porcine | 2014 | Belgium |
| AB924100.1 | G4 | porcine | 2014 | Japan |
| KT820783.1 | G5 | porcine | 2014 | China |
| AB611693.1 | G5 | porcine | 2010 | Japan |
| KC254781.1 | G5 | porcine | 2011 | Brazil |
| JN410646.1 | G11 | porcine | 2007 | Denmark |
| JX470521.1 | G6 | bovine | 2009 | Canada |
| EU548032.1 | G10 | bovine | 2007 | Ireland |
| KP013394.1 | G10 | bovine | 2011 | Iran |
| MH424426.1 | G10 | bovine | 2012 | Iran |
| **VP4 (human)** |  |  |  |  |
| MH291331.1 | P[4] | human | 2017 | Kenya |
| LC514528.1 | P[4] | human | 2018 | Thailand |
| MG652355.1 | P[4] | human | 2016 | Dominican Republic |
| KY748311.1 | P[6] | human | 2011 | Thailand |
| KC579738.1 | P[8] | human | 1980 | USA |
| MF168111.1 | P[8] | human | 2013 | USA |
| KJ659488.1 | P[8] | human | - | USA |
| LC477409.1 | P[8] | human | 2018 | Japan |
| MN529639.1 | P[8] | human | 2018 | China |
| LC491560.1 | P[8] | human | 2018 | Vietnam |
| LC172539.1 | P[8] | human | 2013 | Japan |
| LC468238.1 | P[8] | human | 2014 | Japan |
| LC477396.1 | P[8] | human | 2018 | Japan |
| LC514473.1 | P[8] | human | 2017 | Thailand |
| MN058759.1 | P[8] | human | 2017 | South Korea |
| MT410498.1 | P[8] | human | 2019 | Japan |
| MG996086.1 | P[8] | human | 2016 | Singapore |
| JX435054.2 | P[9] | human | 2007 | Russia |
| MF469343.1 | P[5] | RotaTeq | 2016 | USA |
| GU565044.1 | P[8] | RotaTeq | 1992 | USA |
| MG570048.1 | P[6] | porcine | 2007 | China |
| MK227950.1 | P[6] | porcine | 2015 | Taiwan |
| KR052749.1 | P[6] | porcine | 1975 | USA |
| AB924098.1 | P[6] | porcine | 2014 | Japan |
| AB573872.1 | P[6] | porcine | 2006 | Japan |
| AB573648.1 | P[23] | porcine | 2006 | Japan |
| LC133539.1 | P[5] | bovine | 1989 | Thailand |
| **VP6 (human)** |  |  |  |  |
| KF726068.1 | I1 | human | 2013 | China |
| LC389889.1 | I1 | human | 2009 | Sri Lanka |
| MG670604.1 | I1 | human | 2015 | Dominican Republic |
| MN837282.1 | I1 | human | 2019 | Thailand |
| MN529644.1 | I1 | human | 2019 | China |
| JQ069566.1 | I1 | human | 2007 | Canada |
| LC477438.1 | I1 | human | 2018 | Japan |
| LC477429.1 | I2 | human | 2018 | Japan |
| LC169944.1 | I2 | human | 2014 | Thailand |
| MN401292.1 | I2 | human | 2017 | Czech Republic |
| MT410499.1 | I2 | human | 2019 | Japan |
| LC477426.1 | I2 | human | 2018 | Japan |
| MN577228.1 | I2 | human | 2018 | Russia |
| MG676133.1 | I2 | human | 2015 | India |
| MN206132.1 | I2 | human | 2017 | Russia |
| MN837093.1 | I2 | human | 2017 | Thailand |
| MN837249.1 | I2 | human | 2018 | Thailand |
| KC443404.1 | I2 | human | 2006 | Australia |
| LC001936.1 | I2 | human | 2011 | Japan |
| KJ919729.1 | I2 | human | 2012 | Hungary |
| KR705392.1 | I2 | human | 2010 | Belgium |
| KJ721706.1 | I2 | human | 2005 | Brazil |
| LC477420.1 | I2 | human | 2017 | Japan |
| LC514518.1 | I2 | human | 2017 | Japan |
| MN837271.1 | I2 | human | 2018 | Thailand |
| MN837263.1 | I2 | human | 2018 | Thailand |
| MN837206.1 | I2 | human | 2018 | Thailand |
| MN837254.1 | I2 | human | 2018 | Thailand |
| LC514496.1 | I2 | human | 2018 | Thailand |
| JQ069618.1 | I2 | human | 2009 | Canada |
| LC477421.1 | I2 | human | 2017 | Japan |
| KU550290.1 | I2 | human | 2015 | Spain |
| KF812713.1 | I2 | human | 2011 | South Korea |
| LC133529.1 | I2 | bovine | 1988 | Thailand |
| KJ411437.1 | I2 | human | 2012 | USA |
| LC336590.1 | I2 | human | 2012 | Japan |
| KP198649.1 | I2 | human | 2009 | Italy |
| KP006511.1 | I2 | human | 2009 | Guatemala |
| KC443602.1 | I2 | human | 2008 | Australia |
| KC215501.1 | I2 | vaccine | 1998 | USA |
| LC133551.1 | I2 | bovine | 1983 | USA |
| AB374146.1 | I2 | bovine | 2008 | Japan |
| LC553621.1 | I2 | bovine | 2007 | Japan |
| KC815662.1 | I2 | equine | 1982 | Japan |
| LC074697.1 | I2 | feline | 2004 | Japan |
| KU708258.1 | I2 | roe deer | 2014 | Slovenia |
| **VP6 (animal)** |  |  |  |  |
| KY271812.1 | I2 | human | 2016 | Russia |
| KP006511.1 | I2 | human | 2009 | Guatemala |
| LC491442.1 | I2 | human | 2016 | Vietnam |
| LC477421.1 | I2 | human | 2017 | Japan |
| AB573082.1 | I2 | bovine | 2006 | Japan |
| AB853894.1 | I2 | bovine | 2013 | Japan |
| LC553621.1 | I2 | bovine | 2007 | Japan |
| LC340023.1 | I3 | human | 2014 | Japan |
| JX036369.1 | I3 | equine | 2008 | Argentina |
| AB924099.1 | I5 | porcine | 2014 | Japan |
| EU372799.1 | I5 | porcine | 2009 | Thailand |
| KF303566.1 | I5 | porcine | 2012 | China |
| MK936421.1 | I5 | porcine | 2018 | Spain |
| MN203570.1 | I5 | porcine | 2013 | Slovakia |
| MH238295.1 | I5 | porcine | 2017 | Spain |
| MK936420.1 | I5 | porcine | 2017 | Spain |
| MN203578.1 | I5 | porcine | 2017 | Slovakia |
| KM820727.1 | I5 | porcine | 2012 | Belgium |
| KU739976.1 | I5 | porcine | 2014 | Taiwan |
| KP753126.1 | I5 | porcine | 2007 | South Africa |
| KU887650.1 | I5 | porcine | 2014 | Czech Republic |
| MK597964.1 | I5 | porcine | 2018 | China |
| MK597975.1 | I5 | porcine | 2018 | China |
| MK410285.1 | I5 | porcine | 2018 | China |
| AB924088.1 | I5 | porcine | 2014 | Japan |
| MF139477.1 | I5 | porcine | 2016 | Thailand |
| MH238299.1 | I5 | porcine | 2017 | Spain |

**Table S4**. The number of rotavirus-associated gastroenteritis cases reported in the catchment area in February and Match in 2018 to 2020 (NIID, 2020).

|  | No. of rotavirus-associated gastroenteritis cases | | | |
| --- | --- | --- | --- | --- |
| Week | 2018 | 2019 | 2020 | Average of  2018 & 2019 |
| 7th week | 1 | 0 | 0 | 0.5 |
| 8th week* | 3 | 0 | 0 | 1.5 |
| 9th week | 2 | 3 | 0 | 2.5 |
| 10th week | 5 | 1 | 0 | 3.0 |
| 11th week | 1 | 3 | 0 | 2.0 |
| 12th week* | 2 | 5 | 0 | 3.5 |
| 13th week | 1 | 4 | 0 | 2.5 |
| *The week of water sample collection in 2020. | | | | |

**Reference**

NIID (2020). *Infectious Diseases Weekly Report, Surveillance Data Table* [Online]. National Institute of Infectious Diseases. Available: <https://www.niid.go.jp/niid/en/survaillance-data-table-english.html> [Accessed December 22 2021].

# Supplementary Figures

**Figure S1.** Rarefaction curves for 43 amplicon samples.

**Figure S2.** Rarefaction curves for prevalent genotypes.

**Figure S3.** Concentration of *Rotavirus A* (RVA) in water samples from DWTP E between April 2018 and March 2019.
